# Supplementary material for: Multiparametric Dual-Time-Point [18F]FDG PET/MRI for Lymph Node Staging in Patients with Untreated FIGO I/II Cervical Carcinoma
Source: J Clin Med. 2022 Aug 23;11(17):4943. doi: 10.3390/jcm11174943 (PMC9456388; doi:10.3390/jcm11174943)
Supplement: Supplementary file 1 [file jcm-11-04943-s001.zip › jcm-1843251-supplementary.pdf]

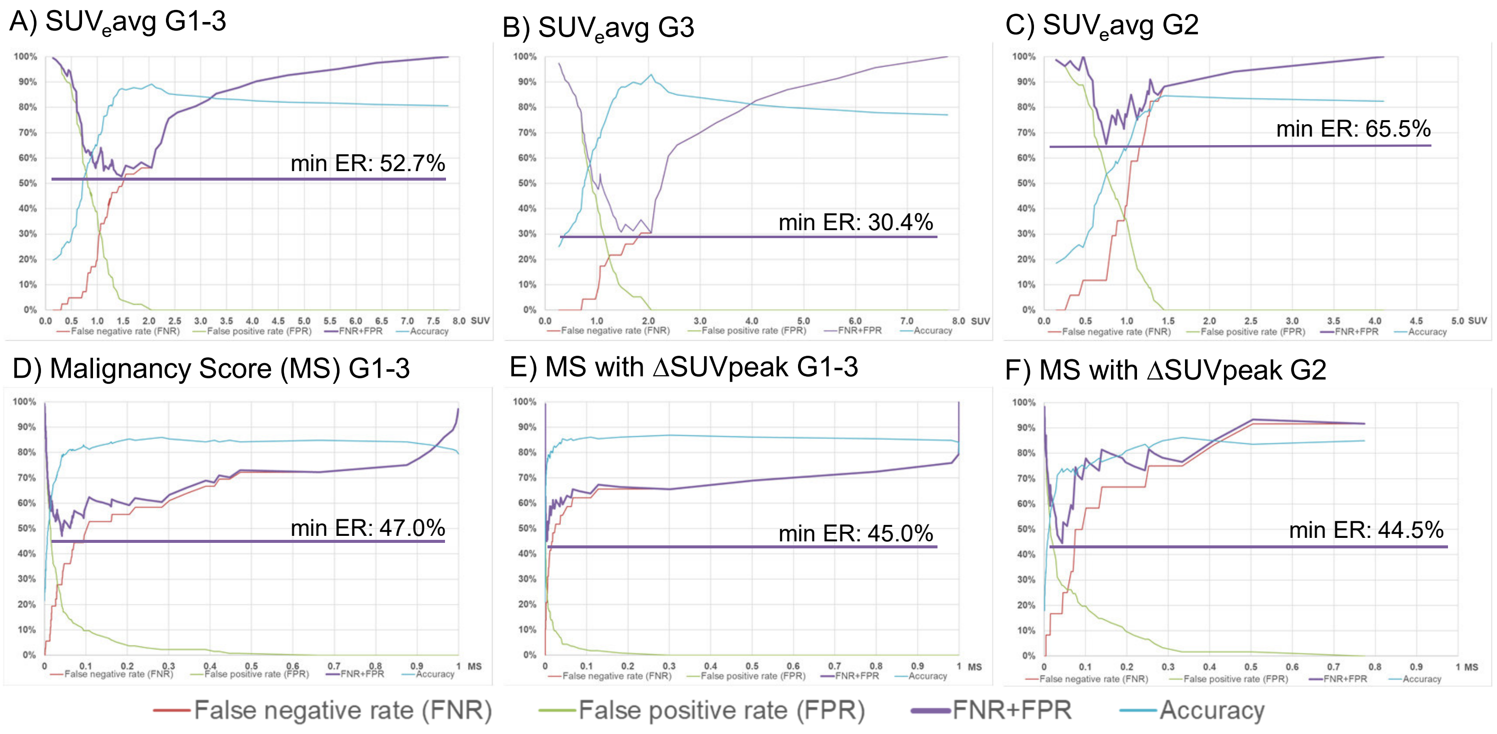

Figure S1. Error rates for the detection of lymph node metastases by single parameter and multiparametric malignancy score in dependence of tumor grading. The multiparametric malignancy score (MS) (**D**) lowers the error rate (ER = false positive rate FNR + false negative rate FPR) about 5 percentage points and stabilizes the ER over a wider range compared to the best single parameter SUV_eavg (**A**). Implementing dual timepoint kinetics (**E**) further enhances this effect and lowers the summed error rate ER by another 2 percentage points compared to the standard MS (**D**). In G2 tumors, this effect is most evident with a significant reduction in summed error rate ER of 21 percentage points from 65.5% (**C**) to 44.5% (**F**). Grade of primary tumor has an huge impact on detectability of lymph note metastases with an doubling of error rates in G2 LNM (C) compared to G3 LNM (**B**) with a sharp increase in FNR starting at an SUV of 1.

Table S1. MRI imaging parameters.

	Slice thickness	Acquisition matrix	In-plane resolution (mm^2)	Repetition time	Echo time	Flip angle	Fat saturation
**Wholebody**							
T2w HASTE cor	5	320x320	1.5625\ 1.5625	1200	91	160	
T2w HASTE tra	5	256x172	0.785\ 0.78125	1200	95	160	
T1w GRE (VIBE) tra	3	384x234	1.3021\ 1.3021	3.95	1.23	10	Dixon fat saturation
DWI (b50/800)	6	128x104	1.7578\ 1.7578	2500	52	90	Water excitation
Post KM: T1w GRE (VIBE)	3	320x195	1.2813\ 1.2813	3.93	1.24/ 2.48	9	Dixon fat saturation
**Pelvis**							
T2w TSE tra.	3	320x320	0.78125\ 0.78125	5760	101	160	
T2w TSE cor.	3	320x310	0.78125\ 0.78125	5880	101	160	
T2w TSE sag.	3	320x310	0.625\ 0.625	5760	101	160	

HASTE: **H**alf fourier **A**cquisition single **S**hot **T**urbo spin **E**cho
VIBE: **V**olume **I**nterpolated **B**reath-hold **E**xamination

Table S2. Survey table of AUC analysis of the dual-time-point PET/MRI parameters for G2 and G3 cervical carcinoma. No lymph node present in G1 tumors.

Parameter	Grading	N	Prevalence	Method	AUC	95%CI	p-value	Std.Error
Size	all	249	17.3%	short-axis	0.741	0.645-0.837	<0.001	0.049
	G2	105	17.1%	short-axis	0.520	0.352-0.679	0.838	0.083
	G3	119	20.2%	short-axis	0.900	0.846-0.961	<0.001	0.030
	all	218	18.8%	SUV$_{e}$avg	0.809	0.728-0.890	<0.001	0.041
SUV early PET				SUV$_{e}$max	0.805	0.723-0.887	<0.001	0.042
				SUV$_{e}$peak	0.807	0.726-0.888	<0.01	0.041
	G2	97	17.5%	SUV$_{e}$avg	0.673	0.529-0.817	0.025	0.073
				SUV$_{e}$max	0.648	0.500-0.796	0.056	0.075
				SUV$_{e}$peak	0.644	0.502-0.786	0.062	0.072
	G3	100	23.0%	SUV$_{e}$avg	0.901	0.820-0.982	<0.001	0.041
				SUV$_{e}$max	0.910	0.837-0.983	<0.001	0.037
				SUV$_{e}$peak	0.909	0.833-0.985	<0.001	0.039
	all	218	18.8%	bpcSUV$_{e}$avg	0.790	0.700-0.880	<0.001	0.046
SUV early PET with bpc				bpcSUV$_{e}$max	0.790	0.700-0.880	<0.001	0.046
				bpcSUV$_{e}$peak	0.761	0.668-0.855	<0.001	0.048
	G2	97	17.5%	bpcSUV$_{e}$avg	0.664	0.511-0.817	0.035	0.078
				bpcSUV$_{e}$max	0.646	0.489-0.802	0.060	0.080
				bpcSUV$_{e}$peak	0.588	0.255-0.431	0.255	0.080

	**G3**	**100**	**23.0%**	**bpcSUV$_e$avg**	**0.866**	**0.767-0.965**	**<0.001**	**0.051**
				**bpcSUV$_e$max**	**0.878**	**0.785-0.971**	**<0.001**	**0.048**
				**bpcSUV$_e$peak**	**0.857**	**0.755-0.958**	**<0.001**	**0.052**
**SUV delayed PET**	**all**	**212**	**17.0%**	**SUV$_d$avg**	**0.741**	**0.640-0.843**	**<0.001**	**0.052**
				**SUV$_d$max**	**0.768**	**0.671-0.866**	**<0.001**	**0.050**
				**SUV$_d$peak**	**0.748**	**0.649-0.846**	**<0.01**	**0.050**
	**G2**	**96**	**17.8%**	**SUV$_d$avg**	**0.570**	**0.411-0.729**	**0.364**	**0.081**
				**SUV$_d$max**	**0.640**	**0.481-0.799**	**0.070**	**0.081**
				**SUV$_d$peak**	**0.581**	**0.426-0.736**	**0.295**	**0.079**
	**G3**	**95**	**20.0%**	**SUV$_d$avg**	**0.883**	**0.788-0.978**	**<0.001**	**0.049**
				**SUV$_d$max**	**0.883**	**0.787-0.979**	**<0.001**	**0.049**
				**SUV$_d$peak**	**0.879**	**0.780-0.978**	**<0.001**	**0.050**
**SUV delayed PET with bpc**	**all**	**212**	**17.0%**	**bpcSUV$_d$avg**	**0.769**	**0.673-8.866**	**<0.001**	**0.049**
				**bpcSUV$_d$max**	**0.799**	**0.709-0.888**	**<0.001**	**0.046**
				**bpcSUV$_d$peak**	**0.768**	**0.676-0.860**	**<0.001**	**0.047**
	**G2**	**96**	**17.8%**	**bpcSUV$_d$avg**	**0.608**	**0.448-0.767**	**0.164**	**0.081**
				**bpcSUV$_d$max**	**0.677**	**0.524-0.830**	**0.023**	**0.078**
				**bpcSUV$_d$peak**	**0.634**	**0.479-0.789**	**0.079**	**0.079**
	**G3**	**95**	**20.0%**	**bpcSUV$_d$avg**	**0.895**	**0.814-0.977**	**<0.001**	**0.042**
				**bpcSUV$_d$max**	**0.905**	**0.836-0.975**	**<0.001**	**0.036**
				**bpcSUV$_d$peak**	**0.874**	**0.787-0.960**	**<0.001**	**0.044**
**ADC**	**all**	**218**	**17.9%**	**ADCmean**	**0.600**	**0.515-0.685**	**0.050**	**0.043**

	G2	86	16.3%	ADCmean	0.580	0.458-0.103	0.343	0.062
	G3	109	22.0%	ADCmean	0.603	0.486-0.721	0.123	0.060
Dual-time-point kinetic - retentionindex	all	179	18.4%	RI-SUVavg	0.544	0.434-0.654	0.429	0.056
				RI-SUVmax	0.608	0.505-0.710	0.054	0.052
				RI-SUVpeak	0.665	0.554-0.777	0.003	0.057
	G2	88	18.2%	RI-SUVavg	0.382	0.234-0.529	0.141	0.075
				RI-SUVmax	0.501	0.352-0.649	0.991	0.076
				RI-SUVpeak	0.525	0.381-0-668	0.758	0.073
	G3	74	23.0%	RI-SUVavg	0.718	0.577-0.858	0.007	0.072
				RI-SUVmax	0.720	0.590-0.851	0.006	0.067
				RI-SUVpeak	0.674	0.530-0.818	0.030	0.073
Dual-time-point kinetic - absolute bpcSUV difference	all	179	18.4%	ΔSUVavg	0.613	0.487-0.739	0.043	0.064
				ΔSUVmax	0.673	0.557-0-790	0.002	0.060
				ΔSUVpeak	0.665	0.554-0.777	0.003	0.057
	G2	88	18.2%	ΔSUVavg	0.422	0.252-0.592	0.330	0.087
				ΔSUVmax	0.542	0.377-0.707	0.600	0.084
				ΔSUVpeak	0.571	0.417-0.725	0.379	0.078
	G3	74	23.0%	ΔSUVavg	0.791	0.644-0.938	<0.001	0.075
				ΔSUVmax	0.793	0.646-0.940	<0.001	0.075
				ΔSUVpeak	0.757	0.605-0.909	<0.001	0.077

bpc= blood pool correction

Table S3. Effect of the tumor grade on PET/MR parameters of lymph node metastases.

Parameter	Grading	N	Mean±SD	p-value
Short-axis diameter (cm)	G2	18	0.48±0.21	<0.01
	G3	24	1.00±0.48	
Sphericity	G2	18	0.58±0.21	0.08
	G3	24	0.67±0.13	
SUV_{e}avg	G2	17	1.01±0.83	<0.01
	G3	19	2.95±2.06	
bpcSUV_{e}avg	G2	17	1.17±0.82	0.03
	G3	19	2.65±1.69	
RI-SUVavg	G2	16	3.83± 29.91	0.03
	G3	17	31.30±37.72	
Δ SUVavg	G2	16	0.08±0.40	0.02
	G3	17	-0.68±0.85	
ADCmean	G2	14	1023±145	0.53
	G3	24	986±216	
